# Seasonal variation of platelets in a cohort of Italian blood donors: a preliminary report

**DOI:** 10.1186/2047-783X-18-31

**Published:** 2013-09-17

**Authors:** Massimo Gallerani, Roberto Reverberi, Raffaella Salmi, Michael H Smolensky, Roberto Manfredini

**Affiliations:** 1 Internal Medicine, Azienda Ospedaliera-Universitaria, Ferrara, Italy; 2 Immunohematological and Transfusional Service, Azienda Ospedaliera-Universitaria, Ferrara, Italy; 3 Department of Biomedical Engineering, The University of Texas at Austin, Austin, USA; 4 Clinica Medica, Azienda Ospedaliera-Universitaria, Ferrara, Italy

**Keywords:** Platelet count, Seasons, Chronobiology, Blood donors

## Abstract

**Background:**

Since available data are not univocal, the aim of this study was to explore the existence of a seasonal variation in platelet count.

**Methods:**

The study was based on the database of the Italian Association of Blood Volunteers (AVIS), section of Ferrara, Italy, 2001–2010. Hematological data (170,238 exams referring to 16,422 donors) were categorized into seasonal and monthly intervals, and conventional and chronobiological analyses were applied.

**Results:**

Platelets and plateletcrit were significantly higher in winter-autumn, with a main peak in December-February (average +3.4% and +4.6%, respectively, *P* <0.001 for both).

**Conclusions:**

Although seasonal variations have been reported for several acute cardiovascular diseases, it is extremely unlikely that such a slight increase in platelet count in winter alone may be considered as a risk factor.

## Background

A broad spectrum of cyclic variations in physiologic and biologic variables exists, depending on both endogenous and environmental effects. Arterial blood pressure rises during winter, due to increased sympathetic activity, decreased loss of fluids and sodium, and elevated blood volume [1]. Again, blood lipids show a significant winter peak independent of age, gender, body mass index (BMI), diet, and physical exercise [1]. As for rheological and hemostatic parameters, a winter peak has been found in young healthy volunteers for plasma viscosity, red blood cell (RBC) deformability, whole blood viscosity, hemoglobin (Hb), hematocrit (HCT), mean corpuscular volume (MCV), fibrinogen, plasminogen activator inhibitor-1 (PAI-1), low density lipoprotein cholesterol (LDL-C), and tryglicerides [2]. On the contrary, no significant seasonal variations were observed for RBC aggregation, total cholesterol, and white blood cell count (WBC). Again, in subjects with and without coronary artery disease, significantly higher values of BMI, glucose, LDL-C, triglycerides, lipoprotein (a), fibrinogen, platelet count (PLT), and lower HDL-C, were found in colder months compared with warmer months [3]. However, reports on platelets are not univocal, and limited to small populations. Thus, we aimed to explore the existence of a seasonal variation in PLT in a large cohort of blood donors in Italy.

## Methods

The study, conducted with the approval of the local institutional committee for human research, was based on the database of AVIS (*Associazione Volontari Italiani Sangue* or Italian Association Blood Volunteers) donors, section of Ferrara, Italy, from January 2001 to December 2010. For each donor, and prior to each donation, accurate medical history was obtained and a visit was arranged (to avoid acute diseases or contraindicating conditions), and a check of Hb level (which could not be <12.5 and <13.5 gr/dl, for women and men respectively). Samples were analyzed by a provincial reference center, with continuous quality controls. A Sysmex XE-2100 analyzer (Sysmex Corporation Production, 1-5-1 Wakinohama-Kaigandori, Chuo-ku, Kobe 651–0073, Japan) was used, which is capable of providing automatic determination of RBC, WBC, reticulocytes, erythroblasts, PLT, mean platelet volume (MPV), platelet distribution curve width (MPV) PDCW, and plateletcrit (PTC). The analyzer was placed in a temperature-controlled environment with air conditioning, to exclude or minimize seasonal fluctuation of the temperature at which the measurement was performed.

Each blood sample was categorized by date into four 3-month intervals (spring: 21 March to 20 June; summer: 21 June to 20 September; autumn: 21 September to 20 December; winter: 21 December to 20 March), and into twelve 1-month intervals, for seasonal and monthly analysis, respectively. The distribution of PLT by season was tested for uniformity in all groups by the *χ*^2^ test for goodness of fit. For monthly analysis, partial Fourier analysis was applied (ChronoLab) [4]. This method selects the harmonic, or the combination of harmonics, that best explain the variance of the time-series data. The percentage of the overall variability of the data about the arithmetic mean that is attributable to the fitted rhythmic function (cosine curve by the method of least squares) estimates the goodness of fit of the approximating model, and the F-test statistic is used to test the zero-amplitude null hypothesis (absence of periodicity). The method calculates the peak and trough time, indicating, respectively, the absolute maximum and minimum values during the year. Significance levels were set at *P* <0.05.

## Results

**Figure 1:**
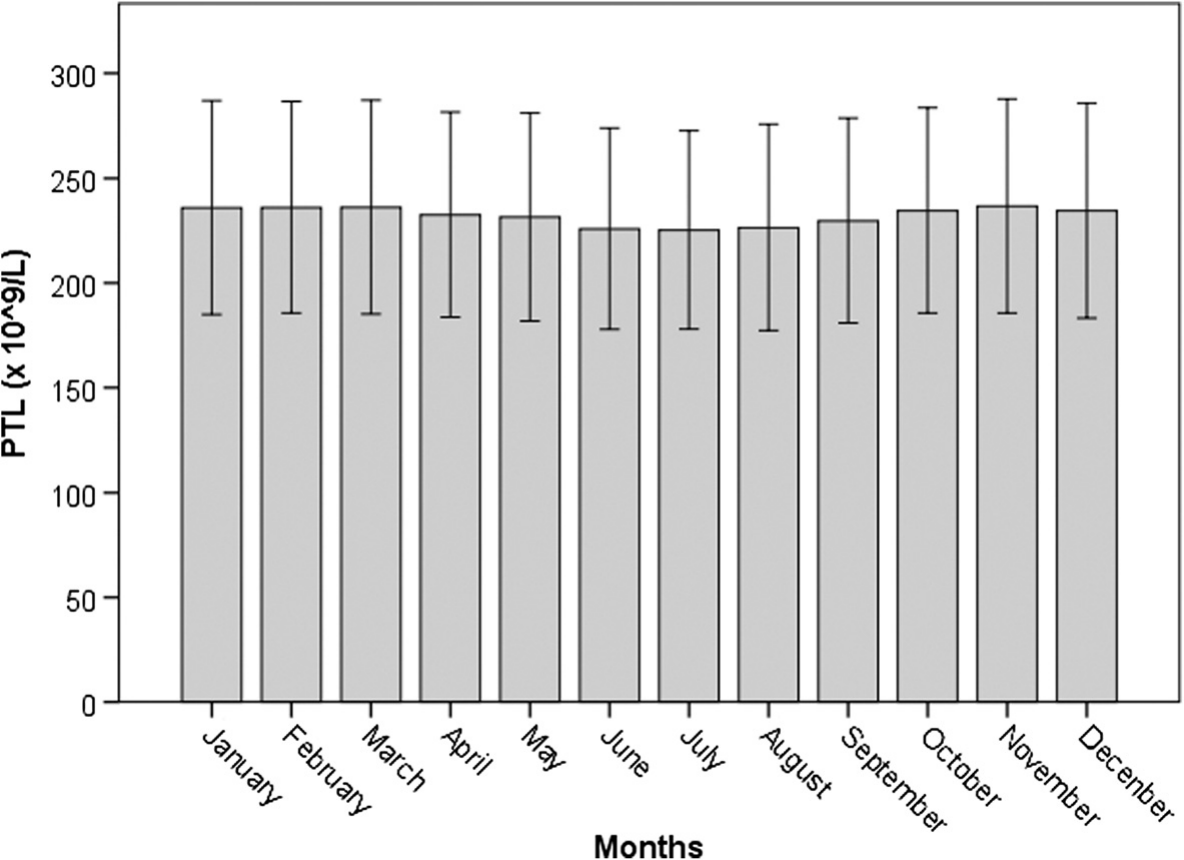
Monthly distribution of platelet count (PLT, media ± SD) in a cohort of blood donors in Italy.

We analyzed 170,238 consecutive samples referring to 16,422 different donors (10,922 men, mean age 43.8 ± 11.5 yrs). Of these samples, 139,638 were from men (mean age 44.3 ± 11.2 yrs), and 30,610 from women (mean age 40.8 ± 12.9 yrs, *P* <0.001). The average number of donations was 9.9 ± 7.3 for men and 5.5 ± 4.6 for women. PLT, PTC, and MPV were parameters considered for calculations. The actual monthly platelet values, media ± SD, are shown in Figure 1.

**Figure 2:**
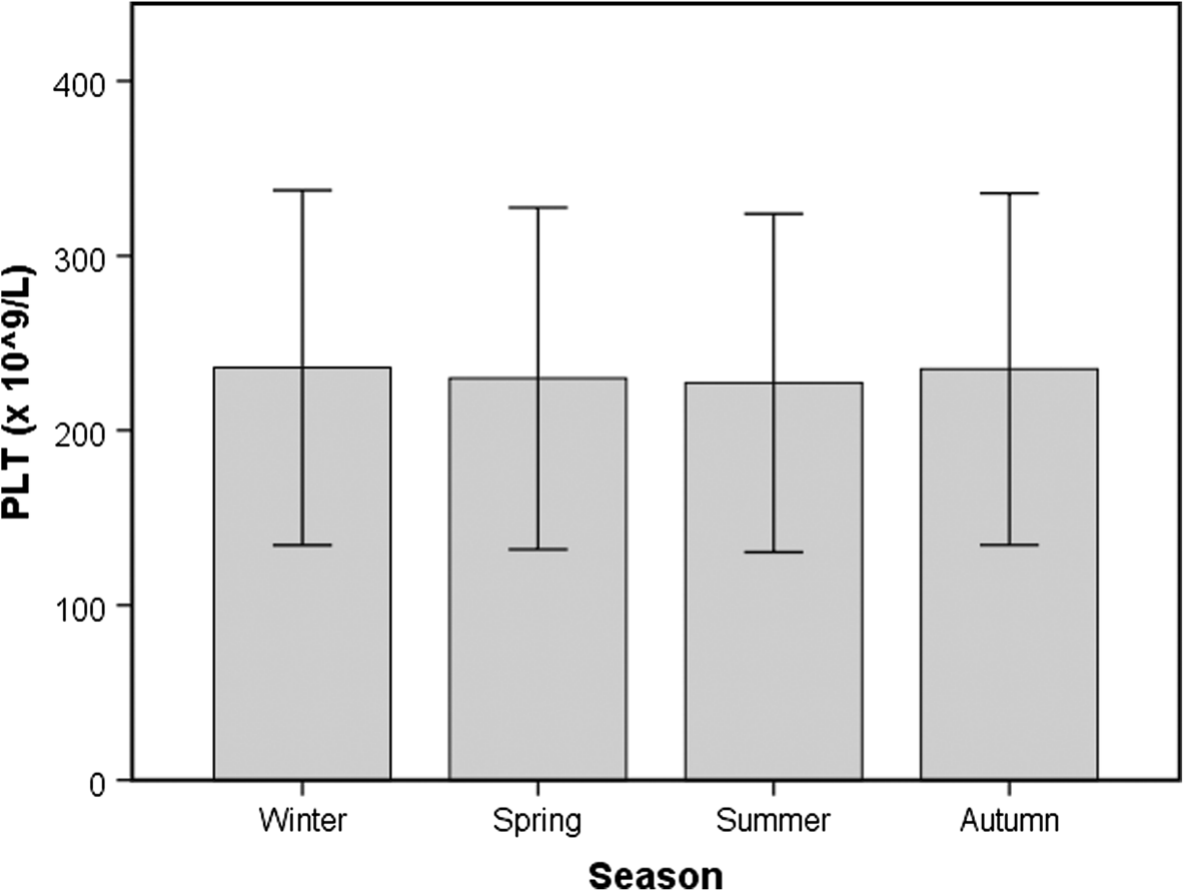
Seasonal distribution of platelet count (PLT, media ± SD) in a cohort of blood donors in Italy.

The seasonal analysis showed higher values of PLT (Figure 2) and PCT in winter to autumn than in summer (average +3.4% and +4.6%, respectively, *P* <0.001 for both). MPV was slightly higher in spring, but such variation was not statistically significant (average +0.4%, *P* = 0.416) (Table 1).

**Table 1:** Seasonal variation of platelet count (PLT), plateletcrit (PTC), and mean platelet volume (MPV) in a cohort of blood donors in Italy

	**Number**	PLT (× 10^9/L)^*^	PTC (%)^**^	MPV (fL)^***^
	**(total = 172,836)**	**average ± SD**	**average ± SD**	**average ± SD**
**Winter**	40,465	235.8 ± 50.8	0.263 ± 0.059	11.43 ± 1.00
**Spring**	43,056	229.7 ± 49.0	0.258 ± 0.057	11.47 ± 0.98
**Summer**	44,208	227.1 ± 48.5	0.251 ± 0.058	11.45 ± 1.00
**Autumn**	45,107	235.0 ± 50.4	0.262 ± 0.061	11.45 ± 0.99

^*^F = 312.08, *P*<0.001; ^**^F = 214.445, *P* <0.001; ^***^F = 1.024, *P* = 0.416.

Chronobiologic analysis yielded a rhythmic variation, with a significant winter peak (December to February) for PLT (total, *P* <0.001; men, *P* <0.001; women, *P* = 0.001; 40 to 59 years, *P* = 0.024), ≥60 years: *P* = 0.001), and PTC (total, *P* <0.001; men, *P* <0.001; women, *P* = 0.001; 40 to 59 years, *P* <0.001; ≥60 years, *P* = 0.003). No significant variation was found for MPD.

## Discussion

An adequate supply of circulating platelets is essential to maintain vascular integrity and to facilitate thrombus formation at sites of vascular injury, and there is evidence that genetic factors, gender and age [5], but also infectious diseases, such as *H. pylori*, may play a role in determining PLT count [6].

Several studies have investigated the existence of seasonal variability in hematological parameters. Data collected in four Asian countries showed lower Hb values during summer in areas with marked seasonal variation in outdoor temperature, but no differences in those with a constant high temperature [7]. As for HCT, a review on 18 studies calculated that it was on average 3% lower (0 to 7%) in summer than in winter [8]. A summer high in HCT deferral rates was reported in a study based on the American Red Cross database [9]. As for PLT, a limited number of studies are available. On one hand, a circadian periodicity was reported for both PLT and platelet aggregability [10]. The highest number of platelets was found in the afternoon, while an increased aggregation was observed in the morning, just when the PAI-1 activity is highest [11,12]. No univocal results on the existence of seasonal variation are available. Findings included reports of PLT increase in the coldest months [13], but also PLT increase in summer, with consistent influences by geographical variation in China [14]. On the other hand, an autumn to winter preference has been reported for a series of acute cardiovascular diseases [15]–[18], and this excess winter risk has been estimated for pulmonary embolism in 14% [19].

The present study shows slight, but significant, seasonal variation in PLT and PTC in healthy subjects, characterized by an autumn to winter peak. This is in agreement with the study by Buckley *et al*., who reported a seasonal variation in PLT count, characterized by a peak during the autumn and winter months and a 2% overall variance [20]. It is possible that the cold temperature may explain these differences, since a mild surface cooling can also increase the packed cell volume (7%) and PLT and MPV (15%) [21], and experimental studies on rats found that diminished PGI2 synthesis/release associated with diminution in the sensitivity of platelets to PGI2 after cooling may explain platelet hyperaggregability [22].

This preliminary study has several limitations: (a) the donors’ database did not include variables potentially influencing PTC, that is, diet or drugs, and (b) no platelet functionality data nor (c) meteorological parameters were available. However, a potential strength derives from the large number of samples available.

## Conclusion

In conclusion, at least in our cohort of healthy donors, a slight but significant increase in PLT count was observed. However, although it is still uncertain whether or not thrombocytosis may be associated with clotting disease [23,24], it is unlikely that such a slight increase alone, at least in healthy subjects, may play a role in the complex relationship between winter and cardiovascular diseases.

## Abbreviations

AVIS: Italian Association of Blood Volunteers; BMI: Body mass index; Hb: Hemoglobin; HCT: Hematocrit; LDL-C: Low density lipoprotein cholesterol; MCV: Mean corpuscular volume; MPV: Mean platelet volume; PAI-1: Plasminogen activator inhibitor-1; PLT: Platelet count; PTC: Plateletcrit; RBC: Red blood cell; WBC: White blood cell count.

## Competing interests

The authors declare that they have no competing interests.

## Authors’ contributions

MG, RR, RM participated in study’s conception and design, interpretation of data, drafting the article and revising it critically for important intellectual content, and final approval. RR and MG handled the original database. RS and MHS participated in the interpretation of data, drafting the article and revising it critically for important intellectual content, and final approval. All authors read and approved the final manuscript.
